# Clinical Features and Prognosis of Herpetic Anterior Uveitis

**DOI:** 10.4274/tjo.92053

**Published:** 2016-06-06

**Authors:** Esra Kardeş, Kansu Bozkurt, Betül İlkay Sezgin Akçay, Cihan Ünlü, Tuğba Aydoğan Gezginaslan, Ahmet Ergin

**Affiliations:** 1 Ümraniye Training and Research Hospital, Ophthalmology Clinic, İstanbul, Turkey

**Keywords:** Elevated intraocular pressure, granulomatous keratic precipitates, herpetic anterior uveitis, iris atrophy, oral acyclovir

## Abstract

**Objective:**

To evaluate clinical features, complications, visual outcomes and treatment modalities in patients clinically diagnosed with herpetic anterior uveitis (AU).

**Materials and Methods:**

We retrospectively reviewed the medical records of 67 patients seen at the Umraniye Training and Research Hospital, Ophthalmology Clinic, Uveitis and Cornea Department from January 2009 to June 2013.

**Results:**

Thirty-seven patients (55.2%) were female and 30 (44.7%) patients were male. The average follow-up period was 12.9 ± 10.6 months (range: 1-45 months). The most common ocular findings were granulomatous keratic precipitates (KPs) (82.2%), corneal involvement (62.6%), iris atrophy (41.7%) and transient elevated intraocular pressure (IOP) (40.2%). Recurrences were observed in 46.2% of the eyes and the median recurrence rate was 1.0 during the follow-up period. Topical steroids and oral antiviral (acyclovir) therapy were applied to all patients during active episodes. Long-term oral acyclovir was used in 29.8% of the patients. Recurrence rates were significantly lower in patients who used oral acyclovir for more than 6 months, whereas complications rates and final visual acuity did not show any difference between groups. Final visual acuity was better than 20/40 in 61.1% of eyes, and visual impairment was due to corneal scarring or cataract formation.

**Conclusion:**

Herpetic AU can present with or without corneal involvement. Granulomatous KPs, iris atrophy and elevated IOP are important clinical findings for the diagnosis of cases without corneal involvement. Long-term oral acyclovir treatment (more than 6 months) and is important to decrease recurrence rates and possible complications. Visual prognosis is favorable in cases without corneal scarring.

## INTRODUCTION

Herpetic anterior uveitis (AU) is a major cause of infectious AU in both developed and developing countries1 and accounts for 5% to 10% of all uveitis cases seen at referral centers.^2,3,4^ Several molecular techniques have been used to identify causative agents, which include herpes simplex virus, varicella zoster virus and cytomegalovirus (CMV).^5,6,7^ However for both accessibility and economic reasons, characteristic clinical findings give the most important clues in the diagnosis of herpetic AU. It is possible to decrease recurrences and prevent vision-threatening complications such as keratitis, glaucoma and cataract in herpetic AU with an early and accurate diagnosis. In the present study, we describe the clinical findings which are helpful in the diagnosis of herpetic AU and analyze its complications, treatment modalities and visual outcomes in a tertiary referral center.

## MATERIALS AND METHODS

We retrospectively analyzed the medical records of 67 patients clinically diagnosed with herpetic AU at the Uveitis and Cornea Service of the Ümraniye Training and Research Hospital from January 2009 to June 2013. Institutional Review Board approval and informed consent from each patient was obtained for the study.

A detailed ocular and medical history was obtained from all patients. A complete ocular examination including best-corrected Snellen visual acuity, slit-lamp biomicroscopy, tonometry and indirect ophthalmoscopy was performed at each visit. Anterior segment photography was performed when indicated. SUN criteria were used for reporting our clinical data.^8^ In patients without corneal disease, the diagnosis of herpetic AU was based on clinical findings such as recurrent unilateral inflammatory attacks in the same eye, acute elevation of the intraocular pressure (IOP) (IOP>22 mmHg) during inflammatory episodes, diffusely distributed or localized granulomatous keratic precipitates (KPs), patchy or sectoral iris atrophy with or without transillumination defects and distorted pupil or spiraling of the iris.^9^

Other causes of infectious or non-infectious uveitis were excluded in patients who did not have corneal involvement or the typical iris atrophy at presentation. A diagnostic workup including complete blood count, liver and kidney function tests, human leukocyte antigen-B27 typing, syphilis serology, chest X-ray, tuberculin skin test and serum angiotensin converting enzyme assay was performed on each patient.

During active AU episodes, patients received oral antiviral treatment (acyclovir), topical anti-inflammatory treatment (topical prednisolone acetate) and/or topical mydriatic agents (tropicamide 1%, cyclopentolate 1% eye drops). Anti-glaucomatous therapy, including topical beta-blockers, alpha-adrenergic agonists and topical or oral carbonic anhydrase inhibitors was initiated in indicated patients. Patients clinically diagnosed with herpetic AU were treated with oral acyclovir 800 mg 3-5 times per day during the active episode and oral acyclovir dosage was maintained at 800 mg daily after the first attack. Once the inflammation was under control, topical corticosteroids were gradually tapered over several months. In order to control inflammation, a long-term, low dose topical steroid (one drop every other day) was continued in patients who showed recurrences after drug cessation. Topical acyclovir ointment was administered to patients with active dendritic keratitis and topical corticosteroids were initiated after epithelial keratitis healed.

Patient demographic features, ocular findings, complications, treatment regimens, recurrences, visual acuity and follow-up period were retrospectively analyzed.

Data were analyzed with SPSS version 22 statistical analysis software (SPSS Inc., Chicago, USA) Student’s t-test was used for comparisons between two groups of parameters showing normal distribution and the Mann Whitney U-test was used for those parameters not showing normal distribution. In the comparison of qualitative data, Fisher’s exact test and Yates Continuity Correction test were used. Levels of significance were accepted as p<0.05.

## RESULTS

Our study included 67 eyes of 67 patients. Thirty-seven patients (55.2%) were female and 30 (44.8%) patients were male. The mean age at presentation was 38.5±18.1 years (range: 3-82 years). The average follow-up period was 14.9±8.6 months (range: 6-45 months). All patients had unilateral involvement. At presentation and during follow-up, 25 eyes (37.3%) had AU without corneal involvement and 42 eyes (62.6%) had AU with corneal involvement. Of these 42 eyes, 25 (59.5%) exhibited stromal keratitis or endotheliitis, 10 (23.8%) had corneal scarring, 5 (11.9%) had epithelial keratitis and 2 (4.7%) had limbal vasculitis. Of the 67 patients, 30 had been diagnosed with uveitis elsewhere and herpetic AU had been diagnosed in 11 (36.6%) of these before referral to our clinic. Corneal involvement was present in all patients who were referred with the correct diagnosis.

**Figure 1:**
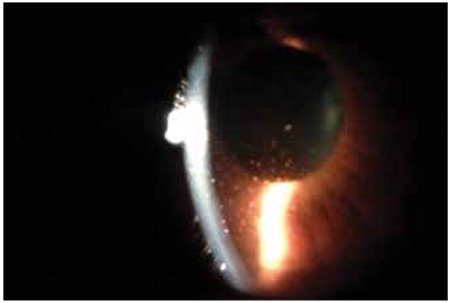
Slit-lamp photograph shows large granulomatous keratic precipitates of a patient with herpetic anterior uveitis

**Figure 2:**
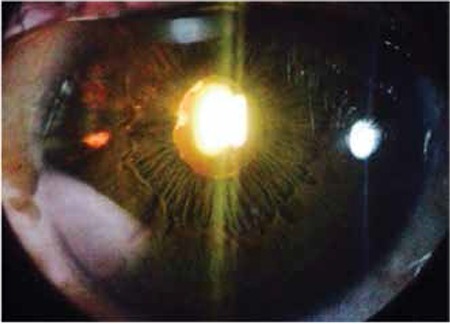
Slit-lamp photograph shows a transillumination defect in the left eye of a patient with herpetic anterior uveitis

During the follow-up period, a total 120 acute episodes (67 at presentation and 53 during follow-up) were recorded in 67 eyes. The clinical findings at each visit were recorded from patient charts. Granulomatous KPs were the most common findings and were recorded in 55 eyes (82%) at least once during follow-up. Granulomatous KPs are medium to large size (mutton fat) (Figure 1) and scattered diffusely or localized under corneal lesions. Fine KPs were observed in the remaining 12 eyes during follow-up. An IOP higher than 21 mmHg during at least one visit was observed in 27 eyes (40.2%). Short-term anti-inflammatory and anti-glaucomatous therapy was applied and IOP was normalized in 19 eyes. Continuous anti-glaucomatous therapy was required in the remaining 8 eyes (11.9%) and 2 of them underwent trabeculectomy. At the final visit, patchy or sectoral iris atrophy was seen in 28 eyes (41.7%) and transillumination defects (Figure 2) were present in 10 eyes (14.9%). Pupil distortion without posterior synechiae was observed in 24 eyes (35.8%). Posterior synechiae were noted in 9 eyes (13.4%) and posterior subcapsular cataract developed in 7 eyes (10.4%) (Table 1).

The median recurrence rate during the follow-up period was 1.0. There was no recurrence in 36 eyes during the follow-up period, while 31 eyes (46.2%) had a total of 53 recurrences. Of the 53 recurrences, 31 (58.4%) exhibited AU, 16 (30.1%) keratouveitis, and 6 (11.3%) keratitis.

Topical corticosteroids were administered to all patients and maintained in 20 patients at very low doses for more than 6 months. The patients with active dendritic keratitis were treated first with topical acyclovir; if uveitis was present, topical steroids were added after the epithelial lesions healed. One patient with dense mutton fat granulomatous KPs received short-term oral corticosteroid therapy. Oral acyclovir treatment was administered to all patients during active keratouveitis and uveitis episodes and was used for more than 6 months in 20 patients (29.8%). Recurrence rates, complication rates, and final visual acuity were compared between patients who were under oral acyclovir treatment for shorter and longer than 6 months. Recurrence rates were significantly lower in patients who used oral acyclovir for more than 6 months, whereas complication rates and final visual acuity did not show any differences between groups (Table 2). Oral acyclovir treatment was discontinued in 8 patients who showed no recurrences during at least 1 year of treatment. Five of these patients experienced recurrences after drug cessation.

Visual acuity at the initial visit was >20/40 in 28 eyes (41.7%), whereas visual acuity at the final visit was >20/40 in 41 eyes (61.1%). Visual acuity less than 20/40 was due to corneal scars in 19 eyes and lens opacity in 7 eyes.

We did not find statistically significant differences in complications and recurrence rates when we compared herpetic AU eyes with or without corneal involvement (p>0.05) (Table 3).

## DISCUSSION

Although herpetic eye disease may occur as a result of primary or recurrent infection, herpetic AU usually occurs as a recurrent infection. Viral replication and immunologic response may both have roles in the pathogenesis of herpetic AU.^10,11^ Herpetic AU is a clinical diagnosis. Confirmation of the diagnosis relies on polymerase chain reaction analyses of intraocular fluids, but in clinical practice characteristic clinical features are important for early diagnosis and treatment.

In the current study, corneal involvement was detected in more than half of the patients with herpetic AU and was present in all of the accurately diagnosed referral patients. This result shows that corneal involvement is an important clinical feature that facilitates the diagnosis. However, the large proportion of referred cases without corneal involvement emphasizes the importance of recognition of the other diagnostic clinical features of herpetic AU. In such cases, other clinical findings of the anterior segment should be indicative of herpetic AU. In this study, the most common ocular findings and complications during follow-up were granulomatous KPs, transient IOP elevation and iris atrophy.

The most frequently encountered ocular finding was granulomatous KPs, which were present in 82% of eyes. These granulomatous KPs were scattered diffusely on the posterior surface of the cornea or remained localized under the corneal lesions. Recently two separate publications from Turkey reported different rates of granulomatous KPs. Nalcacioglu-Yüksekkaya et al.^12^ found a 38% rate of granulomatous KPs, whereas Tugal-Tutkun et al.^13^ found granulomatous KPs in 93% of their patients, which supports our finding.

Inflammation of the trabecular meshwork may lead to a transient increase in IOP. However, long-term use of topical steroids and trabecular meshwork scarring due to chronic and frequent inflammation can cause a persistent increase in IOP. Transient IOP increase was found in 40.2% of our cases and treated with short-term topical anti-glaucomatous therapy. Secondary glaucoma developed in 13.4% of cases and was treated with continuous topical anti-glaucomatous therapy. Glaucoma surgery was performed on two of the secondary glaucoma cases. Different results have been reported from other publications. Van der Lelij et al.^14^ reported elevated IOP in 90% of their cases. However, it is not specified whether this elevation was transient or persistent. Although Tugal-Tutkun et al.^13^ reported transient IOP elevation in 51% of their cases, only 1.8% of them progressed to secondary glaucoma. In a recent Turkish study, secondary glaucoma was found in 31.8% of subjects.^12^ Different approaches in long-term steroid regimens might be an explanation for the variation in the rate of secondary glaucoma in the aforementioned reports.

Patchy or sectoral iris atrophy in herpetic uveitis is associated with transillumination defects. Tugal-Tutkun et al.^13^ reported iris atrophy in 48% of patients and Nalcacioglu-Yüksekkaya et al.^12^ reported it in 49%, both of which are similar to our rate of 41.7%. There are also other uveitic entities associated with iris atrophy. Diffuse iris atrophy causing loss of the corrugated texture of the iris stroma may develop in Fuchs’ Uveitis syndrome (FUS).^15^ The chronic course of FUS and diffusely scattered KPs with stellate extensions may be helpful in the differential diagnosis. CMV-related AU may also cause sectoral iris atrophy with recurrent hypertensive attacks. Unlike herpetic AU, refractory glaucoma tends to develop more commonly in CMV AU.^16^ Distorted pupils are another clinical feature seen in herpetic AU. In the current study pupil distortion was observed in 35.8% of cases. Distorted pupils were reported in 20.3% of cases by Nalcacioglu-Yüksekkaya et al.12 and 25% of cases by Tugal-Tutkun et al.^13^

Oral antiviral agents and topical steroids are the currently used treatment modalities for patients with herpetic AU. The efficacy of prophylactic oral acyclovir is evidenced by a decrease in the number of recurrences of herpetic eye disease.^17^ Sudesh and Laibson^18^ recommended a daily maintenance dose of acyclovir for a minimum of two years after the first uveitis attack. Similarly, in the current study prophylactic oral acyclovir was initiated in all patients after the first uveitis attack. Nearly half (46.2%) of the patients showed recurrences during a mean follow-up period of 12.9 months. However, we found that recurrence rates were significantly lower in patients who used oral acyclovir treatment for longer than 6 months. Topical steroids were also initiated in all patients with uveitis attacks and tapered very gradually to prevent severe rebound attacks. We tried to find a threshold steroid dosage to maintain remission in some patients and used this low dose regimen as a long-term treatment.

Visual prognosis was favorable in spite of the frequent recurrence rate. In our series, the visual outcome of herpetic AU was ≥20/40 in 61.1% of eyes. This is similar to the results reported by Nalcacioglu-Yüksekkaya et al.^12^ Visual impairment in the current study was associated with corneal scars and lens opacity. In addition, the present study reports the outcomes of both primary diagnoses of herpetic AU in our clinic (37 out of 67) and referred patients with uveitis of unknown etiology (30 out of 67). As a result, we could not include the recurrences and survey of referred patients until the diagnosis. This finding may be evaluated as a weak point of the study since the follow-up period and recurrence rate reported might not represent the real-life experience of patients with herpetic AU.

## CONCLUSION

Herpetic AU is usually associated with corneal involvement. However, in patients without corneal involvement, other anterior segment findings are sufficient to make an early and accurate diagnosis. Unilateral and diffusely distributed granulomatous KPs associated with elevated IOP and iris atrophy have a high clinical diagnostic value for herpetic AU. Although the disease has a recurrent nature, visual prognosis is favorable in cases without corneal scarring. Long-term oral acyclovir treatment (more than 6 months) and close follow-up are important to decrease recurrence rates and possible complications.

## Ethics

Ethics Committee Approval: Retrospective study, Informed Consent: It was taken.

Peer-review: Externally and internally peer-reviewed.

## Figures and Tables

**Table 1:**
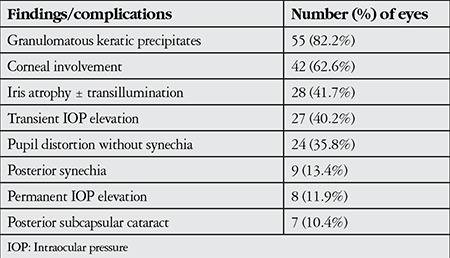
Ocular findings and complications in 67 eyes clinically diagnosed with herpetic anterior uveitis

**Table 2:**
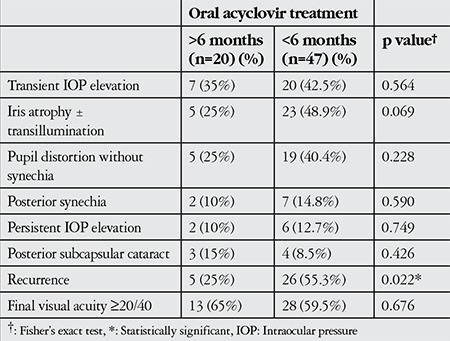
Comparison of recurrence rates, complication rates and visual improvement between patients who were under oral acyclovir treatment for shorter or longer than 6 months

**Table 3:**
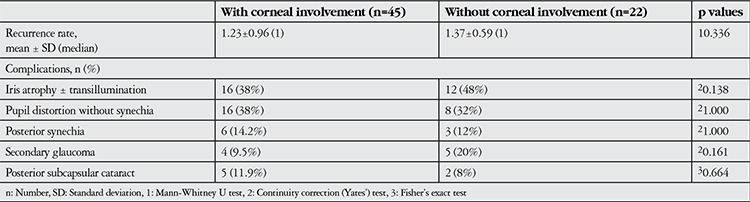
Complications in eyes with or without corneal involvement

## References

[1] Rathinam SR, Namperumalsamy P (2007). Global variation and pattern changes in epidemiology of uveitis. Indian J Ophthalmol.

[2] Cunnigham ET (2000). Diagnosing and treating herpetic anterior uveitis. Ophthalmology.

[3] Gaynor BD, Margolis TP, Cunnigham ET (2000). Advances in diagnosis and management of herpetic uveitis. Int Ophthalmol Clin.

[4] Guney E, Akcay BI, Erdogan G, Unlu C, Akcali G, Bayramlar H (2012). The etiologic features of anterior uveitis in a Turkish population. Clin Ophthalmol.

[5] Schyver I, Rozenberg F, Kestelyn P, Lehoang P, Davis JL, Bodaghi B (2006). Diagnosis and treatment of cytomegalovirus iridocyclitis without retinal necrosis. Br J Ophthalmol.

[6] Usui M, Usui N, Goto H, Minoda H, Rai T (1993). Polymerase chain reaction for diagnosis of herpetic intraocular inflammation. Ocul Immunol Inflamm.

[7] Yamamoto S, Pavan-Langston D, Kinoshita S, Nishida K, Shimomura Y, Tano Y (1996). Detecting herpesvirus DNA in uveitis using the polymerase chain reaction. Br J Ophthalmol.

[8] Jabs DA, Nussenblatt RB, Rosenbaum JT (2005). Standardization of Uveitis Nomenclature (SUN) Working Group. Standardization of uveitis nomenclature for reporting data: results of first international workshop. Am J Ophthalmol.

[9] Siverio Júnior CD, Imai Y, Cunningham ET (2002). Diagnosis and management of herpetic anterior uveitis. Int Ophthalmol Clin.

[10] Liesegang TJ (1999). Classification of herpes virus keratitis and anterior uveitis. Cornea.

[11] Liesegang TJ (1988). Ocular herpes simplex infection: pathogenesis and current therapy. Mayo Clin Proc.

[12] Nalcacioglu-Yüksekkaya P, Ozdal PC, Teke MY, Kara C, Ozturk F (2014). Presumed herpetic anterior uveitis: a study with retrospective analysis of 79 cases. Eur J Ophthalmol.

[13] Tugal-Tutkun I, Otük-Yasar B, Altinkurt E (2010). Clinical features and prognosis of herpetic anterior uveitis: a retrospective study of 111 cases. Int Ophthalmol.

[14] Van Lelij A, Ooijman FM, Kijlstra A, Rothova A (2000). Anterior uveitis with sectoral iris atrophy in the absence of keratitis: a distinct clinical entity among herpetic eye diseases. Ophthalmology.

[15] Jones NP (1993). Fuchs’ heterochromic uveitis: an update. Surv Ophthalmol.

[16] Boxtel LA, Los LI (2007). Cytomegalovirus as a cause of anterior uveitis in immunocompetent patients. Ophthalmology.

[17] Young RC, Hodge DO, Liesegang TJ, Baratz KH (2010). Incidence, recurrence, and outcomes of herpes simplex virus eye disease in Olmsted County, Minnesota, 1976-2007: the effect of oral antiviral prophylaxis. Arch Ophthalmol.

[18] Sudesh S, Laibson PR (1999). The impact of the herpetic eye disease studies on the management of herpes simplex virus ocular infections. Curr Opin Ophthalmol.

